# Inorganic Photoluminescent Microparticles as Identifiers for the Sorting of Lithium‐Ion Battery Cathodes

**DOI:** 10.1002/cssc.202502724

**Published:** 2026-04-18

**Authors:** Simon Ziegler, Christof Strohhöfer, Guojun Gao, Andreas Flegler, Guinevere A. Giffin

**Affiliations:** ^1^ Fraunhofer R&D Center Electromobility Fraunhofer Institute for Silicate Research (ISC) Würzburg Germany; ^2^ Polysecure GmbH Freiburg Germany; ^3^ Institute of Inorganic Chemistry Institute for Sustainable Chemistry & Catalysis with Boron (ICB) Julius‐Maximilians‐University Würzburg Würzburg Germany

**Keywords:** battery recycling, fluorescent marker, inorganic microparticles, tracer‐based sorting

## Abstract

The growing demand for lithium‐ion batteries in the European Union contributes to critical raw material scarcity. This challenge can be addressed by establishing a circular economy, making use of highly efficient and economical recycling processes. The efficiency of these processes depends on high‐purity material streams and thus on the availability of battery data during recycling. While common identifiers could carry this information, they are not suitable for every use case. The present study proposes the use of photoluminescent (PL) microparticles as identifiers for the efficient sorting of battery cathodes. These particles are implemented in the electrodes and can be identified by their unique fluorescence emission signals. This study demonstrates the feasibility of using PL microparticles in the cathode environment and focuses on their practical application in a pre‐industrial pilot sorting facility. The relationship between particle concentration and measurable emission signal is ascertained, and the influence of the PL particles on the electrochemical performance is examined. Highly efficient sorting using three different PL microparticles is demonstrated on a pilot‐scale sorting plant. Finally, an overview of the wide variety of possible identifier codes is provided, which is key to exploiting the potential of this technology.

## 1 Introduction

The increasing demand on lithium‐ion batteries leads to challenges in the availability of critical raw materials for batteries, which is highly dependent on the geological distribution and the geopolitical state of the supply chain [1]. Consequently, the availability of raw battery materials is, particularly for the European Union, a topic of concern [2]. This challenge can be addressed by establishing a battery recycling system which recovers the valuable or critical materials and enables a circular economy [3]. Direct recycling, which preserves the structure and function of the electrode active materials, has significant potential for economically competitive recycling of high value materials like NMC, and enabling economic recycling of low‐cost materials like LFP [4, 5]. Direct recycling is likely to be most effective when combined with cell opening and disassembly to maintain high‐purity material streams, and thus may be less likely to be based on common commercial comminution methods such as cell shredding, which are typical with hydro‐ or pyrometallurgical recycling. To obtain high‐purity material streams, effective sorting processes are required. For the subsequent steps in a direct recycling process chain, the cell chemistry is of particular importance. For example, the conditions for the regeneration processes are typically quite different for layered lithium metal oxides (e.g., LCO, NMC, NCA, …) and olivine lithium metal phosphates (e.g., LFP) [6]. Furthermore, trace impurities, associated with a cell shredding process resulting in a mixed black mass containing the cell casing, electrode components including the active materials, binders, conductive carbon and current collectors, separators and residual electrolyte, also impact the regeneration and the output product [6].

To achieve material consistency during recycling, key information about the batteries and their contents, i.e., battery chemistry, must be available and accessible during the sorting of the recycling process [7]. Kar et al. [8] provides an overview of the challenges in battery sorting and gives a list of possible techniques, including their limitations and advantages. In terms of design‐for‐circularity [9], the use of unambiguous tags attached to the battery, like alphanumerical codes, QR codes or radio‐frequency identification (RFID) tags could provide necessary information about the batteries and their contents [10, 11]. However, these identification systems must be protected from mechanical damage, detachment, soiling, should be both unforgeable and easy to implement in existing manufacturing practices as well as compatible with the disassembly routes. Alternative and advanced technologies include particle‐based systems such as magnetic supraparticles, which have already been demonstrated in the battery environment [12] and inorganic rare‐earth based fluorescent particles for tracer‐based sorting [13]. Particle‐based methods provide new implementation possibilities enabling access to the information and sorting at different stages of battery disassembly during recycling. In all currently reported implementation cases, these markers are not affected by the disadvantages of common tags and can easily be integrated into existing manufacturing processes. The functionality and advantage of fluorescent particles have, i.e., already been demonstrated in plastic sorting and recycling at a technology readiness level of 6 [14, 15, 16, 17].

This work utilizes photoluminescent (PL) microparticles and demonstrates their use for tracer‐based identification and sorting of lithium‐ion battery cathodes. The tracer‐based concept aims to sort cathodes after disassembly of battery cells and before further processing steps such as delamination or comminution. Following their integration into aqueous processed carboxymethyl cellulose‐based nickel cobalt manganese oxide (NCM811) cathodes, the influence of the marker particles on the electrochemical performance in half‐cells and full‐cells, against a lithium‐metal and graphite anodes, respectively, and the detectability of their fluorescent signal within the cathode environment is evaluated. As an important step toward the practical relevance, the reduction of particle content in the cathodes is systematically investigated to establish a correlation between the particle concentration and the measurable emission signal, thus working toward a reliable detection limit. Implementing the marker particles with a concentration as close as possible to the detection limit is further necessary to minimize the impact on the battery energy density. Pre‐industrial scale sorting of marked cathodes was performed on a pilot plant using three different PL microparticles to demonstrate the technological potential of this identification technology.

## 2 Results and Discussion

### 2.1 Fluorescent Particles for Identification of Battery Cathodes

The marking of the electrodes is done using proprietary inorganic microparticles of highly chemically stable, crystalline yttrium oxysulfide from Polysecure GmbH. These microparticles are doped with ytterbium (Yb^3+^) as light‐absorbing sensitizer ions and co‐doped with trivalent lanthanide activator ions (Ln^3+^) to modulate the fluorescence emission. The particles are excited using an infrared (IR) laser at a wavelength of 978 nm [18]. The resulting emission signals are based on two processes occurring within the particles, described as up‐conversion photoluminescence (UC‐PL) and downshifting (DS), also known as anti‐stokes fluorescence and stokes emission, respectively [19, 20]. In the present work, only the UC‐PL signals are used for the particle identification due to the background‐free signal, tailored emission color, and long PL lifetime [20]. To generate a signal variation, emission modulation was performed using holmium (Ho), erbium (Er), and thulium (Tm) as lanthanide co‐dopants, with their characteristic emission spectra in the visible light region, green (maximum at 543 nm) and red (maximum at 674 nm), and in the near‐IR (maximum at 798 nm), respectively [18]. From this point on, these PL microparticles are referred to as YbHo, YbEr, and YbTm, respectively. All of the work was done using the YbHo particles, except for the sorting trials.

The implementation of the marker particles in the electrodes creates new requirements compared to their original application in plastics. Due to the relatively low layer thicknesses of battery electrodes, the particles had to be reduced in size to around 4 µm. The scanning electron microscopy (SEM) images and particle size distribution analysis are shown in Figure S1 a and b, respectively. A homogeneous distribution of the microparticles in the electrodes, as shown in Figure S2, is achieved by adding them directly in a mixing step during the electrode slurry preparation. Thus, the functionality of the electrodes is not expected to be affected by accumulation of the (most likely) redox inactive microparticles or by blocking of the charge transfer pathways, as seen for other particle‐based systems [12].

The measurement of PL particles in the electrode environment requires different measurement parameters than what would be needed for a (light‐colored) plastic substrate. The black electrode materials absorb the excitation light, which is likely combined with a heating effect. This results in a nonreliable decrease of the fluorescence emission over time, likely due to the abovementioned heating effect. The emission fully recovers after a few seconds when the electrode has cooled down. To achieve comparable measurements, the emission was measured within a millisecond time frame after the initial excitation.

### 2.2 Applicability of PL Microparticles in Battery Electrodes

To investigate the marking functionality of the PL microparticles, 5.0 wt% YbHo particles were integrated into nickel cobalt manganese oxide (NCM811) cathodes, to demonstrate a proof of function only. Subsequently, the influence of the PL particles on the electrochemical performance of the electrodes during cycling, along with the effect of cycling on the marker particles, was examined. This emission signal was analyzed at several points during cycling, beginning with the pristine cathodes directly after preparation, continuing through the formed state, and at various points during cycling up to a maximum of 300 cycles. Cycling in the half cells was performed at 1C, after three formation cycles at 0.1C.

**FIGURE 1:**
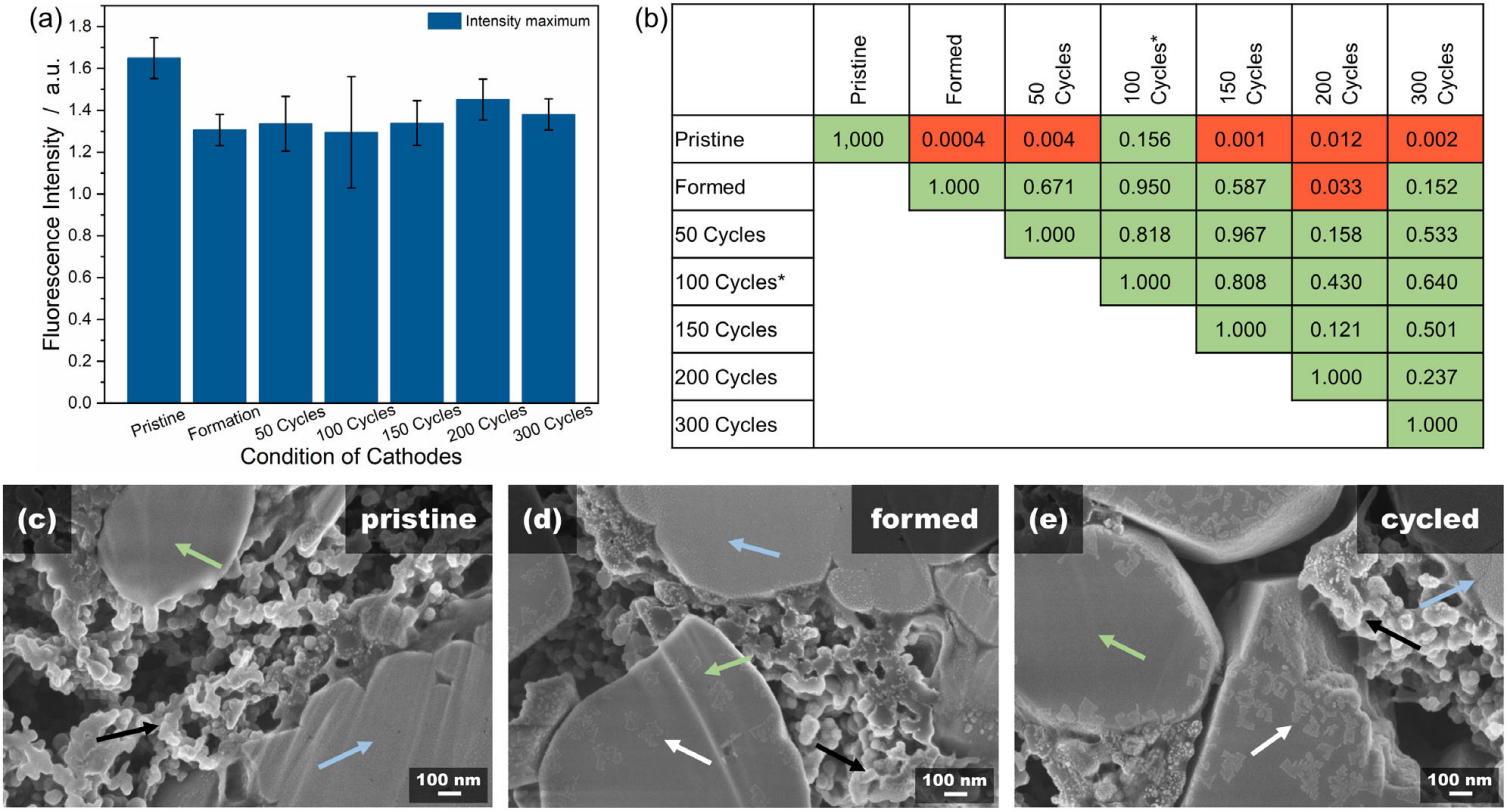
Measurement of the fluorescence intensity of cathodes with 5.0 wt% YbHo marker particles after varying cycle numbers (a) and the corresponding significance analysis matrix of the intensities of the different conditions (b). The red coloring shows a significant (α = 0.05) variation in the fluorescence intensity while the green coloring shows unsignificant variations. (c–e) SEM cross‐section images of representative cathodes in the pristine, formed and cycled conditions. The white arrows show the appearance of a new layer on top of the marker particles (green arrows), which is attributed to a surface layer that may suppress the fluorescence signal intensity. NCM and carbon black are marked with the blue and black arrows, respectively. *Only the three functioning cells were analyzed, compared to five cells, otherwise.

The specific discharge capacity of the half cells, shown in Figure S3 and normalized to the amount of cathode active material, exhibits a steady decrease. The coulombic efficiency (CE) of the cells is always close to 100% not, showing unexpected high or low values during cycling, which could hint at side reactions within the cells. Thus, a typical electrochemical behavior of the cells can be seen. Visible fluctuations at high cycle numbers result from lithium anode reactions [21]. The functionality of the YbHo particles after cycling was assessed by measuring the fluorescence emission signal of the cathodes recovered from the cells. After opening, the electrodes were separated and washed with dimethyl carbonate to remove any electrolyte residue. Figure 1a shows the fluorescence signal intensity for all samples, which reveals an apparent difference between the pristine cathodes and the electrodes recovered from the cells. The specific discharge capacity of the half cells, shown in Figure S3 and normalized to the amount of cathode active material, exhibits a steady decrease. The CE of the cells is always close to 100%, not showing unexpected high or low values during cycling, which could hint at side reactions within the cells. Thus, a typical electrochemical behavior of the cells can be seen. Visible fluctuations at high cycle numbers result from lithium anode reactions [21]. The functionality of the YbHo particles after cycling was assessed by measuring the fluorescence emission signal of the cathodes recovered from the cells.

After opening, the electrodes were separated and washed with dimethyl carbonate to remove any electrolyte residue. Figure 1a shows the fluorescence signal intensity for all samples, which reveals an apparent difference between the pristine cathodes and the electrodes recovered from the cells. The average decrease in the fluorescence intensity is measured to be around 18%. A closer look at the intensity differences between the various cathodes is conducted by significance analysis using a two‐sided t‐test. Figure 1b shows the result matrix which confirms a significant variation of the signal intensity between the pristine and cycled cathodes, indicated by the red coloring. The analysis matrix shows almost no significant variations in the fluorescence intensity of the cycled samples regardless of the number of cycles experienced.

To determine the cause of the decrease of fluorescence intensity, the YbHo particles within the electrodes were examined with SEM. The SEM cross‐section images of representative cathodes in the pristine, formed and cycled state are displayed in Figure 1c,d and e, respectively. The NCM, carbon black and the PL microparticles can be identified in all three images (marked with the blue, black and green arrows, respectively). In addition, a surface layer (marked with a white arrow) is visible on the surface of the marker microparticles after the electrode has been recovered from the cell environment. This layer is more distinct with the cycled cathode than for the formed cathode. As the coverage of the particle by this surface layer is more significant, this layer can be described as a cathode electrolyte interphase (CEI). A visually similar layer is also present on the surface of the NCM particles. The CEI forms on the entire cathode and consists mainly of oxidation products of the electrolyte [22]. Li et al. even showed that, in the presence of fluoroethylene carbonate, which was used as an electrolyte additive in this study, this interphase consists of a structured, multilayer system of an amorphous polymer matrix and lithium oxide grains [23]. Consequently, the CEI layer is expected to partially block or absorb the excited laser light or the emission fluorescence signals. Although the CEI layer typically grows during cycling [24, 25], no further significant reduction of the fluorescence intensity after the formation could be measured, at least during the 300 cycles, which was the set limit of cycling in this study.

### 2.3 Reduction of Particle Concentration Toward Practical Relevance

One of the main challenges with the implementation of design for recycling/circularity strategies is gaining industrial acceptance. Minimizing the penalty in terms of energy density, power density, lifetime, cost, etc. of implementing such technologies is key. To reduce the impact of introducing an electrochemically inactive component in the electrodes, the concentration of PL microparticles must be reduced to a minimum while maintaining their function as identifiers for the sorting of electrodes. In this section, the effects of the reduction in concentration of YbHo particles from 5.0 wt% to 0.5 wt%, with intermediate steps of 2.5 wt% and 1.0 wt%, are examined. The samples are named according to the concentration of marker particles integrated, e.g., YbHo‐0.5 contains 0.5 wt% marker.

**FIGURE 2:**
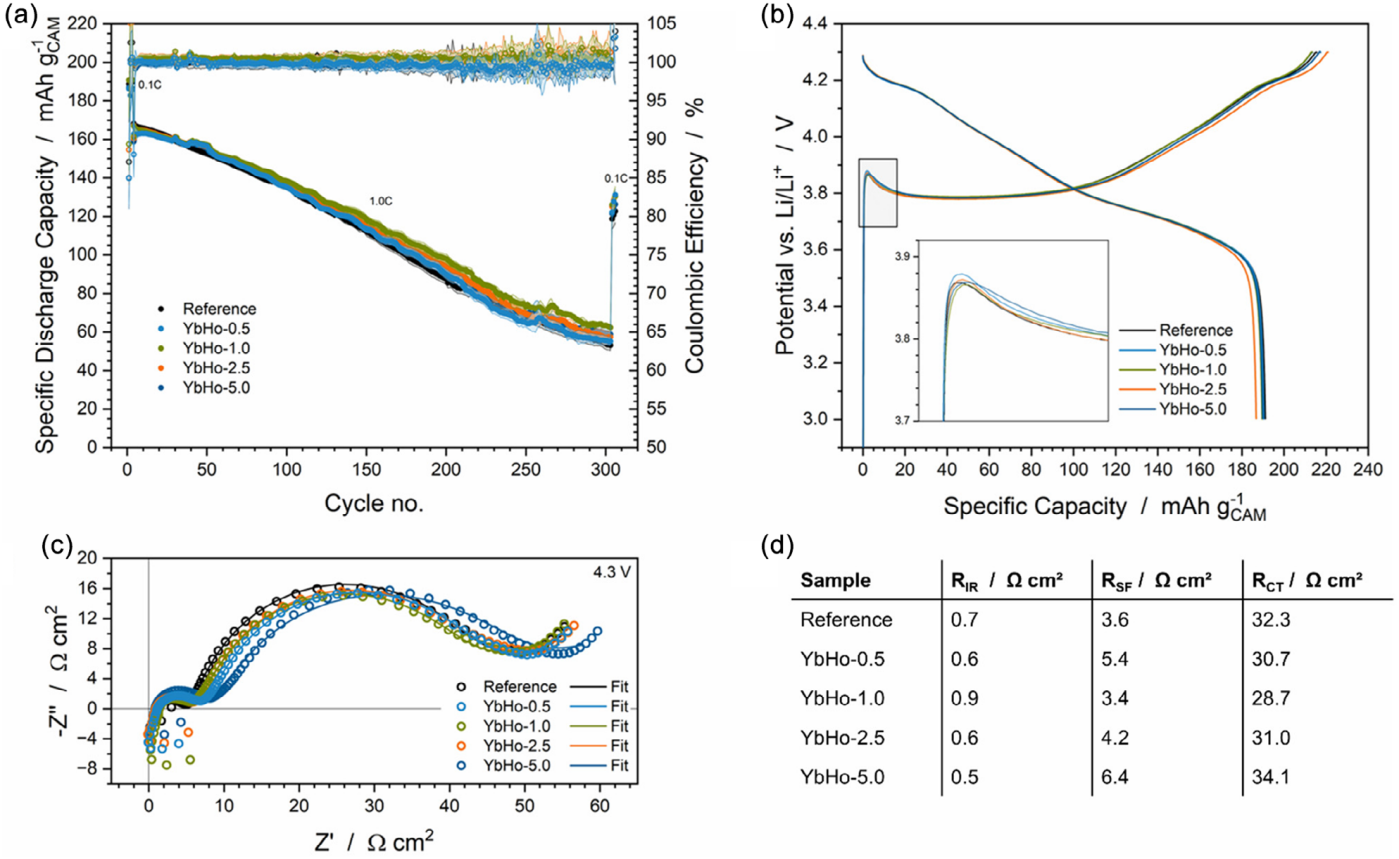
Electrochemical data from half cells with cathodes, including varying concentrations of YbHo marker particles from 0.5 wt% to 5.0 wt%. Specific discharge capacity and CE over 300 cycles (a) and the voltage profiles of the first charge and discharge, with a focus on the initial overpotential (inset b). Nyquist plots of the EIS data at 4.3 V (c) with resistance values after fitting with the equivalent circuit shown in Figure S4 (d).

The electrochemical data for the cathodes with different concentrations of marker particles are shown in Figure 2. There are only minor and likely insignificant differences in the specific discharge capacity over 300 cycles for all samples, including the reference without particles. The same is true for the initial discharge capacities, with an average value of around 190 mAh g_CAM_^−1^. Only the YbHo‐2.5 sample exhibits a small deviation, with a value around 187 mAh g_CAM_^−1^, which is likely within the error range of manual electrode coating and cell preparation. The capacity retention after 300 cycles at 1C (CR; capacity of first cycle at 1C compared to last cycle at 1C) of the cells containing the markers is slightly higher, with values of 32% for the reference and 35%, 34%, 37%, and 34% for the marked electrodes YbHo‐5.0, YbHo‐2.5, YbHo‐1.0, and YbHo‐0.5, respectively. Given that these measurements are all done in half cells, the fading seen is most likely associated with the lithium metal electrode rather than the cathodes. This is also evident from the high fluctuations in the CE, starting at about 200 cycles, as mentioned before. Figure 2b shows the initial charge and discharge curves of representative cells, focusing on the initial overpotentials. The curves are almost identical, with differences in the initial polarization on the order of ca. 13 mV. As there is no clear trend in the initial polarization with marker concentration and as all of the cell preparation steps are done by hand, including cleaning the surface of the lithium metal, these differences are unlikely to be caused by the marker particles in the cathode.

Electrochemical impedance spectroscopy (EIS) measurements were performed at 4.3 V to determine the influence of the microparticles in the cathodes on the cell impedance. The EIS data is shown in the Nyquist plots in Figure 2c, along with the corresponding resistance values obtained by fitting the data with the equivalent circuit shown in Figure S4 [26, 27]. The high frequency intercept can be attributed to the ohmic resistance of the cell (R_IR_). The R_IR_ is low, between 0.5  and 0.9 Ω cm^2^, for all cells. The high‐frequency semi‐circle is associated with the surface film resistance (R_SF_) [28, 29]. This contribution is influenced by both the solid electrolyte interphase (SEI) on the lithium metal surface and by the CEI, as can be seen on the cathode surface in the SEM images in Figure 1 [30]. The resistance R_SF_ ranges between 3.4  and 6.4 Ω cm^2^. The low‐frequency semi‐circle is attributed to the charge transfer resistance [29, 31, 32, 33] (R_CT_) and also covers only a small range between 28.7 and 34.1 Ω cm^2^. For all three resistance values, there is no distinct trend with concentration that can be discerned from the data. Thus, the impedance data suggest that there is no significant difference in the reference and the electrodes containing the marker particles, and also within the concentration series. These results seem to suggest that the differences in the resistance are likely more associated with differences due to the manual fabrication of the electrodes of the cell than the fluorescent particles. In the context of marker integration, there is no indication that the particles have a significant impact on the electronic conductivity of the electrodes or on the surface film resistance (CEI and SEI). As such, it can be concluded that the marker particles have no significant impact on the electrochemical performance of the electrodes, at least within the tests conducted here. As such, the main consideration in determining the appropriate concentration of marker particles for practical relevance is the concentration that still allows effective sorting. Naturally, this concentration should be kept as low as possible to minimize the loss of energy density due to the particle mass/volume. Table S1 shows the calculated loss of gravimetric and volumetric energy density for the cathodes containing 5.0 wt% to 0.5 wt% of PL particles.

**FIGURE 3:**
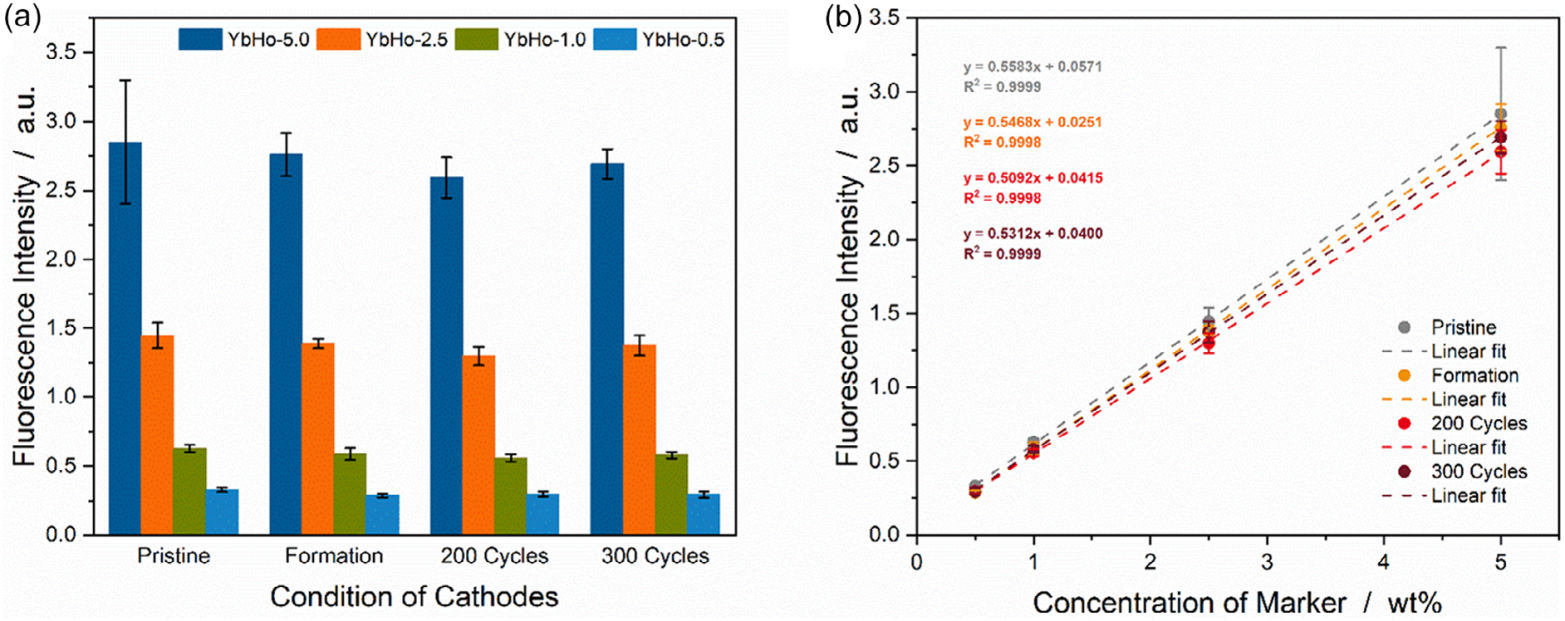
Fluorescence intensity analysis of cathodes with varying concentrations of marker particles and at different points during cycling. The fluorescence intensity for the pristine and cycled cathodes with marker particle concentrations of 5.0 wt% to 0.5 wt% (a). The fluorescence intensity as a function of the marker concentration for the different conditions, with the corresponding linear regression line (dotted) and the calculated coefficient of determination (b).

Efficient sorting depends on the detectability of the marker particles and thus their fluorescence response within the cathodes. The emission signal of the microparticles was investigated both as a function of concentration and the different conditions experienced by the cathodes (pristine and formed states, as well as after 200 and 300 cycles). The data is shown in Figure 3. It is worth noting that all concentrations of PL microparticles could be measured, even with only 0.5 wt% of YbHo, without approaching the detection limit. As expected, the measurements show decreasing intensities for lower concentrations of PL microparticles, as well as slight variations in the signal intensity for the different cathode conditions. The significance analysis matrices are shown in Table S2 to Table S5. A significant signal change is still seen in some cases between the pristine state and the cathodes that experienced electrochemical reactions, as described above. Nevertheless, this effect is less pronounced and not observed for all marker concentrations.

The correlation between the concentration of marker particles and the measured signal intensities has also been analyzed. The data and the associated linear regression are shown in Figure 3b. The slopes of all four data sets, i.e., the intensity data from a single cathode condition, are very similar and in the range of 0.51–0.56. The signal intensity falls linearly with concentration, as supported by the high coefficients of determination, R^2^, which is greater than or equal to 0.9998 for all data sets. Based on these results, this data can function as a calibration curve, which allows the fluorescence signal to be estimated at other concentrations. Using the detector sensitivity limit, an appropriate microparticle concentration can be selected, which allows the amount of microparticles within the cathodes to be reduced to a minimum until the fluorescence emission reaches the detection limit.

**FIGURE 4:**
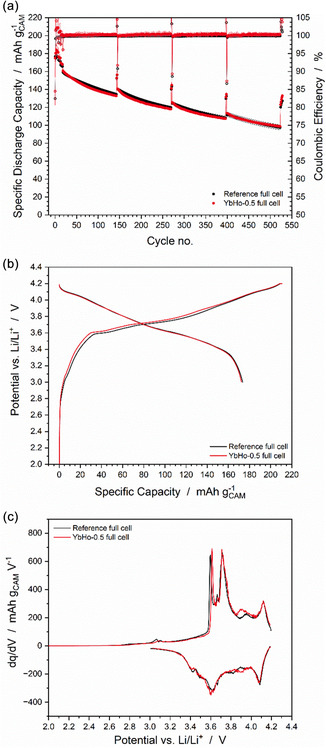
Electrochemical data for reference full cells as well as cells including 0.5 wt% YbHo. The specific discharge capacity and CE (a), the first cycle charge and discharge curves (b) and the differential capacity plots of the first cycle (c).

As the lowest concentration of marker particles investigated (0.5 wt%) could be easily detected in the cathode environment, this concentration was chosen for testing in full cells with a graphite anode. Full cell testing is important to demonstrate practical relevance. In lithium metal half cells, the supply of lithium in the cell is almost endless. As such, other mechanisms, such as overpotential at the lithium metal lead to capacity fading. In contrast, in full cells, the amount of active lithium, i.e., the lithium inventory, is limited to that provided by the cathode. Thus, all lithium inventory that is lost to side reactions in the cell, either wanted reactions such as SEI/CEI formation in the initial cycles [34, 35] or unwanted reactions such as electrolyte decomposition [36], directly affects the cell lifetime. The full cell cycling data for YbHo‐0.5 full cells in red, compared to the references in black is shown in Figure 4. The difference between the cells with and without the marker particles is almost negligible and within the range of the error. Both samples demonstrate initial capacities of around 160 mAh g_CAM_^−1^ at 1C. The capacity decreases to approximately 100 mAh g_CAM_^−1^ after 500 cycles, resulting in a CR of 62.5%. The negligible differences are further displayed in Figure S5 showing minor variations in the charge and discharge curves of the check‐up cycles at 0.1C. Slight differences between the YbHo‐0.5 and the reference are revealed in the initial charge and discharge curves at 0.1 C in Figure 4b and in the differential capacity plot of the same cycles in Figure 4c. The YbHo‐0.5 full cells show a slightly higher polarization during charge in the mid state of charge range. This slight increase in polarization is also evident in the differential capacity plots, particularly in the charging process, where the reaction peak at around 3.6 V is shifted to higher potential of approximately 20 mV. Furthermore, the cell containing the marker has a higher first cycle irreversible capacity, evident from the difference in the charge and discharge capacity. Nonetheless, these small effects have little impact on the capacity retention at the end of cycling.

### 2.4 Cathode Sorting at Pilot Plant‐Scale

**FIGURE 5:**
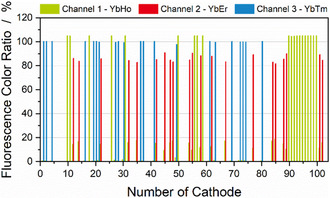
Cathode sorting with a pre‐industrial sorting machine based on fluorescence emission using three fluorescent markers. Based on the measured color ratio, cathodes are assigned to the corresponding fractions. 100% purity was achieved in all fractions.

The results shown above have demonstrated that the PL microparticles have a neglible impact on the electrochemical performance of the cells, and can still be detected with the lowest concentration tested, even after cycling, in the cathode environment. However, to more fully demonstrate the practical relevance toward an industrial application, the marked cathodes were sorted on a pre‐industrial scale sorting plant, as displayed in Figure S6. For these tests, the three different types of PL microparticles were integrated into larger cathodes, 3  x 5 cm. These cathodes (20 YbHo, 21 YbEr and 21 YbTm) were then mixed with 40 unmarked reference cathodes. The electrodes were manually fed into the sorting plant in a random order. A three‐detector system, one for each signal color, was used to distinguish the electrodes. The initial fluorescence intensities of all the cathodes are shown in Figure S7. Due to the fact that the YbEr‐samples emit light in the green and red color fields, and the markers have different photoluminescence quantum efficiencies [20], color ratios were calculated via a proprietary software to distinguish between the marker types. This allows the differentiation of the markers with the cathodes and thus sorting of the cathodes, as displayed in Figure 5. Each bar in the figure represents one marked cathode, with the color of the bar indicating either the green fluorescence of YbHo or the red fluorescence of YbEr, or the near IR fluorescence displayed in blue of YbTm. The signal detection and analysis allowed 100% of the electrodes to be sorted correctly. The use of larger cathodes, containing 0.5 wt% particles, produced a signal‐to‐noise ratio (S/N) of around 30. This noise is primarily due to shot noise in the electronics and does not scale with signal intensity. Assuming that the intensity scales linearly with concentration of the fluorescence marker, as shown in Figure 3, reducing the concentration by a factor of 2.5 would bring the S/N ratio to approximately 12. The sorting line has shown reliable determination of marker fluorescence above a threshold of 10. Consequently, the resulting concentration of microparticles could be reduced from 0.5 wt% to around 0.2 wt% based on the results of this study. The further reduction of the marker amount would minimize the impact on gravimetric and volumetric energy density and thus positively influence the feasibility of this sorting technology.

## 3 Conclusion

This study has demonstrated the use of fluorescent microparticles as identifiers in battery cathodes to enable effective sorting. The PL microparticles could be easily integrated into NMC cathodes with a short additional mixing step. The markers had a negligible effect on the electrochemical performance even at a relatively high concentration (5 wt%). The marker particles are electrochemically inactive under the conditions tested within this study, and thus, the concentration used should be small to minimize the effect on the energy density. The PL microparticles could be detected within the black electrodes even at a relatively low concentration (0.5 wt%). A small but statistically significant reduction in the emission fluorescence intensity was observed for the cathodes recovered from the cell after testing (even after only the formation cycles) in comparison to the pristine cathodes. This reduction is likely associated with the CEI, which is visible on the surface of the particles and the cathodes. Nonetheless, the signal intensities remain above the detection limit and are thus measurable. The sorting of cathodes containing three different types of PL microparticles was demonstrated on a pre‐industrial scale sorting pilot plant. The cathodes could be differentiated, which enabled a sorting efficiency of 100% as well as a purity of 100%. The data provided here a clear proof of concept of the marker technology at practically relevant concentrations.

Although only three marker particles were used in this study, a high variety of PL microparticle identifiers is possible. For example, the known markers may be mixed in certain ratios, additional transition metals may be used to dope the microparticles and modulate the emission signal, and the combined detection of the UC and NIR emissions can be implemented to create a greater number of unique emission lines per marker [14, 20]. In the context of direct recycling and its importance for low‐cost cathodes, transferability to other CAM chemistries should be validated.

## 4 Experimental Section

### 4.1 Marker Preparation

The fluorescent particles were milled via a mild milling process to smaller particle sizes to enable better implementation in the cathodes. One kilogram of marker particles, 2 kilograms of 2 mm glass balls, and 2 kilograms of water were mixed in a 6 L round‐bottom flask. The mixture was milled for 2 h at 200 rpm. The milled markers were then heat‐treated for 4 h at 300°C and sieved.

### 4.2 Preparation of Battery Cells

#### 4.2.1 Cathode Manufacturing

NMC811 cathodes were prepared with a water‐based process. Initially, a mixture of 6.5218 g deionized water and 0.2174 g carboxymethylcellulose binder (Sigma–Aldrich Chemie GmbH) was stirred for approximately 14 h at 750 rpm on a Vibramax. Subsequently, 5.0000 g of NMC811 (Gelon LiB Group) and 0.2174 g of Super C65 conductive carbon (C‐NERGY, Imerys S.A.) were added, and the mixture was stirred in a Speedmixer at up to 2400 rpm. Finally, the PL marker particles were added to the slurry with an additional stirring step at 2000 rpm for 2 min. The slurries were coated on 20 µm aluminum foil (Korff AG) by the doctor blade method with a coating velocity of 5 mm/s and a gap of 225 µm. The electrode sheets were dried at 80°C for 30 min prior to calendaring to a porosity of 50% and punching electrodes in a rotary press, either discs with a 16 mm diameter or a 5  x 3 cm rectangular format. Finally, the electrodes were dried at 110°C for 12 h under a vacuum of approximately 50 mbar. The electrodes had an areal capacity of ∼2.0 mAh cm^−2^. The PL marker particles were ytterbium‐doped oxysulfide‐based crystals, co‐doped with holmium, erbium, or thulium, as described in the text above. The concentrations of marker particles were 0.5 wt% to 5 wt% relative to the rest of the electrode composite content. The reference electrodes were prepared in the same way, but without the additional stirring step at 2000 rpm.

#### 4.2.2 Fabrication of Pouch Cells

Battery cells were fabricated with the 16 mm‐sized cathodes in half‐cell and full‐cell configurations and a Celgard 2500 (Celgard LLC) separator. Half cells were prepared with a lithium metal electrode (Gelon Lib Group Co., Ltd., China) laminated on a copper current collector. The lithium metal was cleaned by scraping it with a ceramic knife to remove the surface layer. The layers were placed between two stainless steel spacers to apply pressure on the cell stack, and the pouch cell was filled with 200 µL of 1 M LiPF_6_ in EC:DMC (1:1 m/m) with 5 wt% FEC electrolyte (E‐Lyte Innovations GmbH) before sealing at 50 mbar. Five cells were fabricated for each sample. Full cells were made with commercial graphite anodes with an areal capacity of 2.4 mAh cm^−2^ (CustomCells Holding GmbH) and were filled with 200 µL 1 M LiPF_6_ in EC:EMC (3:7 m/m) with 2 wt% VC electrolyte (E‐Lyte Innovations GmbH). Three full cells were made with each sample.

### 4.3 Analytical Methods and Characterization

#### 4.3.1 SEM

SEM was performed on the particle powders using a scanning electron microscope ZEISS Supra 60VP (Carl Zeiss Microscopy GmbH) equipped with an inlens SE‐II detector. A thin layer of powder was dispersed on an adhesive carbon disc and sputtered with a thin layer of silver to aid conductivity. SEM imaging of the cathodes were performed on a ZEISS Ultra 55 (Carl Zeiss Microscopy GmbH). The cross‐sections were prepared by argon‐ion milling on a SM‐0910 cross section polisher (JEOL GmbH).

#### 4.3.2 Particle Size Distribution

Particle size distribution measurements were conducted with a Fritsch Analysette 22 Next in the range of 0.01–3800 µm. The measurements were performed 3 times, and the values were determined as the mean of the three measurements.

#### 4.3.3 Galvanostatic Cycling

Electrochemical testing of the pouch cells was performed on an electrochemical workstation MCT 10‐06−96 ME (Digatron Power Electronics GmbH) in a temperature chamber at 25°C (Memmert GmbH & Co. KG). In case of the half cells, after a resting period of 12 h, three formation cycles at C/10 in a voltage range of 3.0 – 4.3 V were performed. Subsequently, the cells were cycled for 50–300 cycles at 1C, prior to finishing the measurement with three cycles at C/10. Full‐cell measurements were conducted with a 24 h rest period before the formation at C/10 (3 cycles) between 3.0 and 4.2 V. Afterwards, a rate test was performed at C/10, C/5 and C/2 in a voltage range of 2.7–4.2 V, with 5 cycles at each rate, prior to starting the long‐term cycling at 1C. The cells were cycled for 500 cycles in total with two check‐up cycles at C/10 after every 125 cycles. The full‐cell testing was finished with five cycles at C/10. The cells were charged with a constant‐current constant‐voltage procedure (CCCV) and discharged in a constant‐current mode (CC). The CV step was terminated at a current of C/20.

#### 4.3.4 EIS

EIS measurements were performed using a VMP300 potentiostat (BioLogic GmbH). The potentiostatic impedance spectra were obtained at 4.3 V with an amplitude of 5 mV over the frequency range of 10 mHZ to 6 MHz. Prior to the impedance measurements, the half cells were formed as described above for the cycling tests. The cells were held at 4.3 V for 3 h to ensure steady state.

#### 4.3.5 Fluorescence Analysis and Sorting

Fluorescence from the cathodes was analyzed in a fluorescence measurement system by Polysecure GmbH, using a laser with a wavelength of ∼ 976 nm for excitation of the PL particles and detection optics with three detectors, one for each possible emission wavelength of the particles used. Optical filters are placed in front of each detector to selectively measure the green (∼545 nm), the red (∼660 nm) and the IR (∼800 nm) emissions for the holmium, erbium and thulium doped particles, respectively. The fluorescence emission intensity is always measured as the integral of a short timespan after excitation and corrected for background. For measurements with only one type of marker particle, the specific detector signal was analyzed. When sorting samples with different marker particles, which was carried out in a sorting pilot‐plant, the fluorescence ratio was used to discriminate between the cathodes. To prevent false signal analysis, e.g., due to scattered light, a signal intensity threshold value of 1500 counts was set.

To analyze the fluorescence intensity between different samples based on significance variations, the intensity values were compared in two‐sided T‐tests for distributions with different variances. The significance level was set to 0.05 for these tests. The results were arranged in matrices for easier comparison and values below and above the significance level were highlighted in red and green, respectively, as shown in Figure 1b.

## Supporting Information

Additional supporting information section can be found on online in the supporting information section.

## Funding

This work was supported by Bundesministerium für Forschung, Technologie und Raumfahrt (03XP0393B and 03XPB0393D).

## Conflicts of Interest

The authors declare no conflicts of interest.

## Supporting information

Supplementary Material
