# Supplementary material for: Inorganic Photoluminescent Microparticles as Identifiers for the Sorting of Lithium‐Ion Battery Cathodes
Source: ChemSusChem. 2026 Apr 18;19(8):e202502724. doi: 10.1002/cssc.202502724 (PMC13091073; doi:10.1002/cssc.202502724)
Supplement: Supplementary file 1 — Supplementary Material [file CSSC-19-e202502724-s001.pdf]

Supporting Information

Inorganic Photoluminescent Microparticles as Identifiers for the Sorting of Lithium-Ion Battery Cathodes

Simon Ziegler [a], Christof Strohhöfer [b], Guojun Gao [b], Andreas Flegler [a], Guinevere A. Giffin *[a,c]

[a] Fraunhofer R&D Center Electromobility, Fraunhofer Insitute for Silicate Research (ISC), Neunerplatz 2, D-97082 Würzburg

[b] Polysecure GmbH, St. Georgener Str. 19, 79111, Freiburg, Germany

[c] Institute of Inorganic Chemistry, Institute for Sustainable Chemistry & Catalysis with Boron (ICB), Julius-Maximilians-University Würzburg, Am Hubland, 97074 Würzburg, Germany

*Corresponding authors:

E-mail: guinevere.giffin@isc.fraunhofer.de (G. A. Giffin)

Supplementary Results

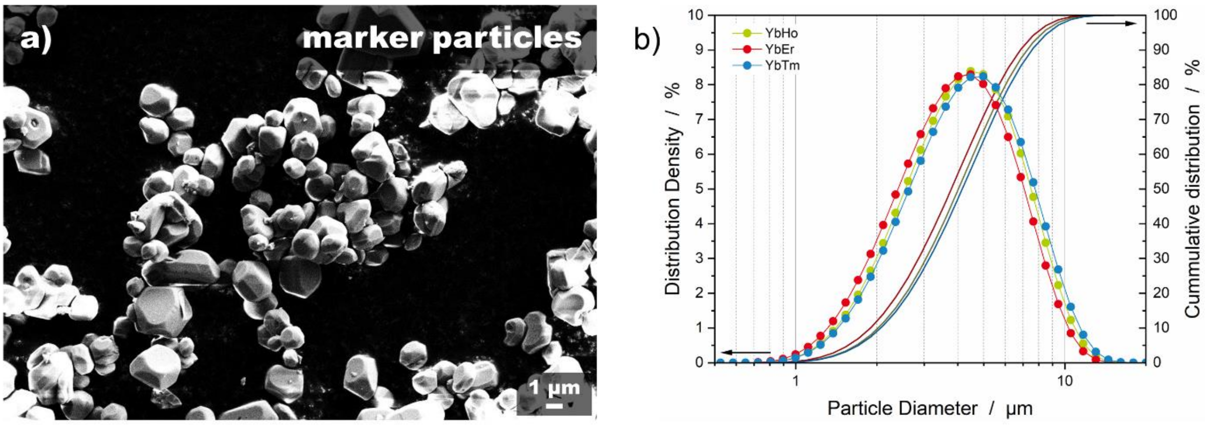

Figure S1: SEM overview image of the holmium-doped yttrium oxysulfide particles as representative images for all of the marker particles used a) and the particle size distribution curves of the doped marker particles shown by their distribution density and related cumulative distribution for YbHo, YbEr and YbTm in green, red and blue, respectively. The D50 values are 4.0 µm for YbHo, 3.8 µm for YbEr and 4.2 µm for YbTm.

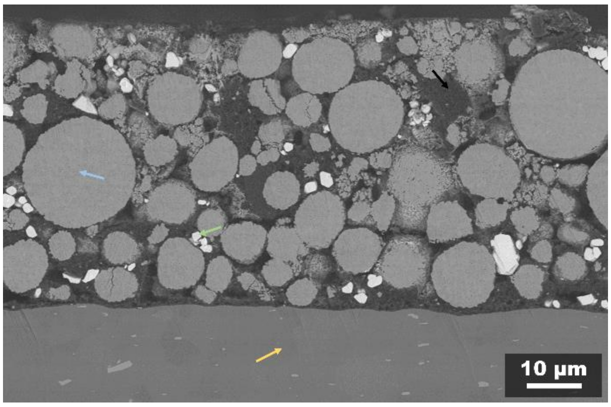

Figure S2: SEM cross-section image of a representative cathode with marker particles. In the backscattered electron detector image, the NCM particles, carbon black and the aluminum current collector are marked with the blue, black and yellow arrows, respectively. The green arrow shows the marker particles which are homogeneously distributed in the cathode.

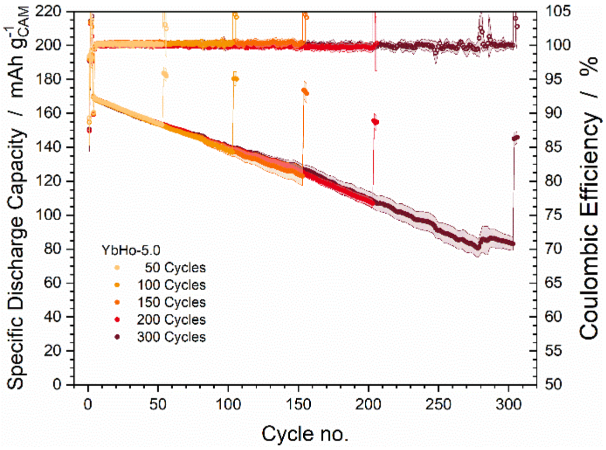

Figure S3: Specific discharge capacity and coulombic efficiency of half cells containing cathodes including 5.0 wt% YbHo marker particles. The capacity data is the average of five cells for each sample, except the 100 cycle cells, where the data is the average of the three functioning cells.

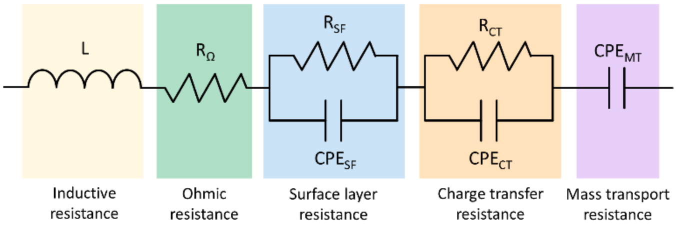

Figure S4: Equivalent circuit used for the fitting of the measured impedance data of the reference half cells and the half cells with marker particles in the cathodes.

Table S1: Calculated loss of energy density at the cathode electrode level related to the percentage of PL marker particles within the cathodes. The calculation was done using same values for the electrode mass, electrode area, porosity, aimed electrode density and the specific capacity of NCM811. The integrated marker particles thus change the mass of cathode active material and electrode density, resulting in a change of electrode thickness.

Percentage of marker [wt%]	5.0	2.5	1.0	0.5	0.2[a]
Loss of mass energy density [%]	4.8	2.4	1.0	0.5	0.2
Loss of volume energy density [%]	3.9	2.0	0.8	0.4	0.2

[a] Proposed minimal usable amount.

Table S2: Significance analysis of fluorescence intensities of cathodes with 0.5 wt% YbHo marker particles in different conditions.

**0.5 wt%**	Pristine	Formation	200 Cycles	300 Cycles
Pristine	1.000	0.002	0.017	0.025
Formation		1.000	0.344	0.595
200 Cycles			1.000	0.794
300 Cycles				1.000

Table S3: Significance analysis of fluorescence intensities of cathodes with 1.0 wt% YbHo marker particles in different conditions.

**1.0 wt%**	Pristine	Formation	200 Cycles	300 Cycles
Pristine	1.000	0.119	0.003	0.018
Formation		1.000	0.254	0.759
200 Cycles			1.000	0.209
300 Cycles				1.000

Table S4: Significance analysis of fluorescence intensities of cathodes with 2.5 wt% YbHo marker particles in different conditions.

**2.5 wt%**	Pristine	Formation	200 Cycles	300 Cycles
Pristine	1.000	0.235	0.022	0.204
Formation		1.000	0.037	0.718
200 Cycles			1.000	0.125
300 Cycles				1.000

Table S5: Significance analysis of fluorescence intensities of cathodes with 5.0 wt% YbHo marker particles in different conditions.

**5.0 wt%**	Pristine	Formation	200 Cycles	300 Cycles
Pristine	1.000	0.688	0.280	0.488
Formation		1.000	0.125	0.456
200 Cycles			1.000	0.270
300 Cycles				1.000

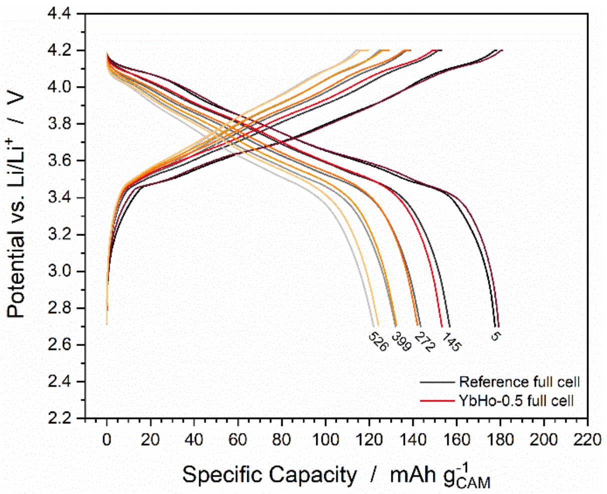

Figure S5: Charge and discharge curves of representative full cells for the reference and with marked cathode. Every second cycle of the check-up cycles and in the beginning and end of cycling at C/10 is displayed with its individual cycle number.

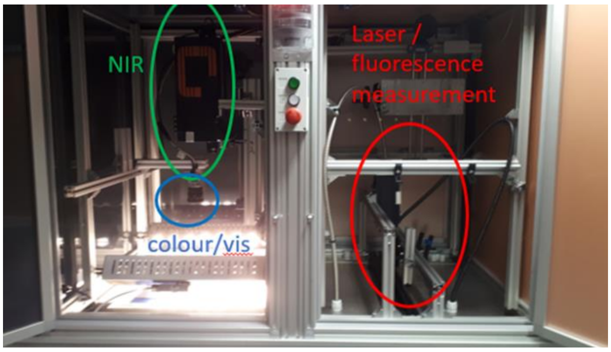

Figure S6: Detection unit of the pilot sorting plant. The detection unit is mounted on a conveyor belt which transports the samples with 1 m/s. In addition to the equipment for the measurement of marker fluorescence, it contains near infrared and color measurement capabilities, which were not used in the current study. The fluorescence measurement was performed with a 980 nm laser as excitation source, beam-shaped into a line to cover the whole width of the conveyor belt, and fiber-coupled optics which collects the fluorescence signal and conveys it to the photodiodes with three different color channels. Color discrimination is achieved with the help of a color-sensitive beam splitter.

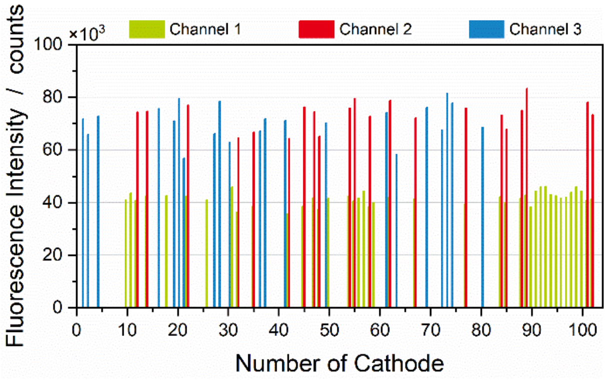

Figure S7: Fluorescence intensity analysis by the three detector signals for each sample cathode going through the sorting plant.
